# Phylogenetic classification of human papillomavirus genotypes in high-grade cervical intraepithelial neoplasia in women from a densely populated Brazilian urban region

**DOI:** 10.1590/S1516-31802009000300003

**Published:** 2009-10-06

**Authors:** Denise Rocha Pitta, Luis Otávio Sarian, Elisabete Aparecida Campos, Sílvia Helena Rabelo-Santos, Kari Syrjänen, Sophie Françoise Derchain

**Affiliations:** 1 MSc. Biologist, Department of Obstetrics and Gynecology, Universidade Estadual de Campinas (Unicamp), Campinas, São Paulo, Brazil.; 2 MD, PhD. Assistant professor, Department of Obstetrics and Gynecology, Universidade Estadual de Campinas (Unicamp), Campinas, São Paulo, Brazil.; 3 PhD. Pharmacist and assistant professor, School of Pharmacy, Universidade Federal de Goiás (UFG), Goiânia, Goiás, Brazil.; 4 MD, PhD, FIAC. Associate professor, Department of Oncology and Radiotherapy, Turku University Hospital, Turku, Finland.; 5 MD, PhD. Associate professor, Department of Obstetrics and Gynecology, Universidade Estadual de Campinas (Unicamp), Campinas, São Paulo, Brazil.

**Keywords:** Human papillomavirus 11, Human papillomavirus 16, Human papillomavirus 18, Human papillomavirus 6, Genotype, Cervical intraepithelial neoplasia, Cancer, Polymerase chain reaction, Papillomavirus 11 humano, Papillomavirus 16 humano, Papillomavirus 18 humano, Papillomavirus 6 humano, Genótipo, Neoplasia intra-epitelial cervical, Câncer, Reação em cadeia da polimerase

## Abstract

**CONTEXT AND OBJECTIVE:**

Differences in human papillomavirus (HPV) types may correlate with the biological potential and invasion risk of high-grade cervical intraepithelial neoplasia (CIN 2 and CIN 3). The objective of this study was to determine the relationship between different combinations of HPV types and CIN severity.

**DESIGN AND SETTING:**

Cross-sectional study, at Universidade Estadual de Campinas (Unicamp).

**METHODS:**

Cervical samples from 106 women treated due to CIN 2 (18) or CIN 3 (88) were examined for specific HPV genotypes using Roche Linear Array® (LA-HPV). The proportions of CIN 2 and CIN 3 in groups of women infected with the HPV phylogenetic groups A7 and A9 were compared. Three groups were formed: women with single infections; multiple infections; and the whole sample.

**RESULTS:**

Multiple infections were detected in 68 samples (64.7%). The most frequent high-risk genotypes detected (single/multiple) were HPV 16 (57.1%), HPV 58 (24.7%), HPV 33 (15.2%), HPV 52 (13.3%), HPV 31 (10.4%), HPV 51 (7.6%) and HPV 18 (6.6%). Women without infection with HPV species Alpha 9 were less likely to have CIN 3 than were their Alpha 9 HPV-infected counterparts. HPV 16 and/or HPV 18, with or without associations with other viral types, were more frequently found in women with CIN 3 than in those with CIN 2.

**CONCLUSIONS:**

The severity of high-grade CIN may be aggravated by the presence of HPV types included in the Alpha 9 phylogenetic classification and by infections including HPV 16 and 18, singly or in combination with other HPV genotypes.

## RESUMO

**CONTEXTO E OBJETIVO:**

Diferentes tipos de papilomavírus humano (*human papillomavirus*, HPV) podem ser correlacionados com a capacidade biológica e risco de invasão das neoplasias intra-epitelial de alto grau cervical (NIC 2 e NIC 3). O objetivo deste estudo foi determinar a relação de diferentes tipos de HPV com a gravidade da NIC.

**TIPO DE ESTUDO E LOCAL:**

Estudo transversal na Universidade Estadual de Campinas (Unicamp).

**MÉTODOS:**

Foram avaliados os genótipos específicos de HPV da amostra cervical de 106 mulheres com NIC 2 (18) ou NIC 3 (88), utilizando Roche Linear Array® (LA) HPV *genotyping assay*. Foram comparadas as proporções de NIC 2 e NIC 3 em grupos de mulheres infectadas com tipos de HPV dos grupos filogenéticos A7 e A9. Três grupos foram formados: mulheres com infecção simples; infecção múltipla; e infecção simples e múltipla.

**RESULTADOS:**

Infecções múltiplas foram detectadas em 68 (64,7%) das amostras. Os genótipos de alto risco mais frequentemente detectados em infecção simples ou múltipla foram HPV 16 (57,1%), HPV 58 (24,7%), HPV 33 (15,2%), HPV 52 (13,3%), HPV 31 (10,4%), HPV 51 (7,6%) e HPV 18 (6,6%). A probabilidade de mulheres com NIC 3 serem infectadas com HPV que não da espécie Alfa 9 era menor do que com os tipos de HPV da espécie Alfa 9. HPV 16 e ou 18, associado ou não com outros tipos virais eram mais frequentemente encontrados nas mulheres com NIC 3 do que naquelas com NIC 2.

**CONCLUSÃO:**

A gravidade da NIC de alto grau pode ser aumentada pela presença de tipos de HPV incluídos na classificação filogenética Alfa 9 e por infecções que incluem HPV 16 e 18 combinados ou não com outros genótipos de HPV.

## INTRODUCTION

Persistent infection with high-risk types of human papillomavirus (HPV) is known to be a unifying risk factor for the development of cervical intraepithelial neoplasia (CIN) and invasive carcinoma.^1^^,^^2^^,^^3^^,^^4^ CIN is classified into three grades, based on progressive spreading of atypical cells from the proliferative layers to the full thickness of the epithelium.^5^ Although CIN 2 and CIN 3 represent high-grade lesions of the cervix, they are heterogeneous in their potential for progression to invasive cancer.^6^

Current evidence indicates that differences in HPV types may correlate with the biological potential and invasion risk of CIN lesions.^2^^,^^7^^,^^8^ Recent data show that the HPV types found in CIN 2 are different from those observed in CIN 3, and CIN 2 frequently contains HPV types that are not commonly found as single types in invasive cancers.^9^ This could suggest that invasive cancer is unlikely to be the end point for such CIN 2 lesions.^9^^,^^10^

Genital HPV types are classified as Alpha papillomavirus genera. The species within these genera are closely related in terms of phylogenesis. Despite having distinct genomic sequences, they show identical or very similar biological or pathological properties. Along with the type species HPV 16, species 9 also includes HPV types 31, 33, 35, 52, 58 and 67. Along with the type species HPV 18, species 7 also includes HPV types 39, 45, 59, 68, 70 and 85.^11^

Controversy exists regarding possible competition or synergy of individual types in multiple infections. Some natural history studies have suggested that women who are already infected present greater risk of acquiring new HPV types than do those who are HPV-negative.^12^^,^^13^ An alternative interpretation of these findings might be that more than one HPV type is transmitted simultaneously, and their sequential detection could be a consequence of replicated life cycles that are asynchronous and only occasionally overlap.^14^ These life cycles might be interdependent.^15^ Multiple HPV types may be associated with a risk of progression that exceeds the risk due to single-type infections. In a recent study, there was evidence that this risk increased with the cumulative number of HPV types, in most combinations, and these associations seemed particularly strong over the short term.^16^

## OBJECTIVE

In this report, our prime focus was to address the question of heterogeneity of HPV types as a possible source of biological variation in CIN 2 and CIN 3. To add new information to the growing body of literature in women with these lesions, we aimed to evaluate the distribution of single and multiple infections of different HPV types in women with high-grade cervical intraepithelial neoplasia (CIN 2 and CIN 3), and to compare the prevalence of different HPV types in CIN 2 and CIN 3, in view of the phylogenetic classification of the virus.

## METHODS

### Type of study and setting

The patient sample for this cross-sectional study comprised 106 non-consecutive women who underwent large loop excision of the transformation zone (LLETZ) to treat CIN 2 or 3, between February 2001 and April 2004.

The study was carried out at the colposcopy clinics of Universidade Estadual de Campinas (Unicamp), Brazil, a public health institution dedicated to comprehensive care for women.

### Sample

The sample size for this study was estimated according to the relationship n = (p*q)/E ^*2*^ , where *n* is the sample size, *p* is the estimated prevalence of the condition in affected women (in the present case, the prevalence of HPV in high-grade CIN is as high as 95%^16^), and *q* is the prevalence of the condition in non-affected subjects. The overall prevalence of highly oncogenic types of HPV in an urban female population (mean age: 33 years) without high-grade CIN has been estimated as 6.2%.^16^*E* is the standard error, set to 2.5% for the present calculations. Therefore, we estimated a sample size of roughly 94 women.

The women in this study were selected after referral to Unicamp for specialized treatment for high-grade squamous intraepithelial lesions (HSIL). They were invited to enter the study at the time of their pretreatment visit (i.e. their first visit after being accepted for treatment). One of the researchers was always present at these visits and, after explaining the study protocol and ethical concerns, made the invitation. All the patients who agreed to be enrolled in the study gave their written signed consent.

At the pretreatment visit, all of the women were interviewed to obtain clinical, social and demographic data. A complete gynecological examination was performed, with collection of endocervical specimens for HPV testing, followed by colposcopic examination of the cervix. The decision to perform diathermal conization was based on the referral cytology and the clinical/colposcopic configuration of the cervix. The study was approved by the local Ethics Committee (Protocol #CEP 309/2004).

### Procedures

#### Histology

The histological samples consisted of 106 diathermal conization specimens. This material was fixed in 10% phosphate buffered formalin and then was embedded in paraffin and stained with hematoxylin and eosin. The samples were analyzed according to the World Health Organization’s criteria.^17^ In the present series, only cases diagnosed as CIN 2 (n = 18) or CIN 3 (n = 88) were included. The patients’ mean age was 34.08 years (90% central range: 17.5 to 73.6 years). The ages of the women with CIN 2 (mean age: 32.01 years; 90% central range: 17.4 to 59.5 years) and CIN 3 (mean age: 34.05 years; 90% central range: 17.3 to 73.6 years) were similar (P = 0.32).

### Main measurements

#### HPV detection

#### DNA extraction

For HPV typing, cervical cells were collected using a Digene™ cervical brush and were then shaken in universal collection medium (UCM). An aliquot of 200 μl of UCM was sampled (Digene Corporation, United States) and centrifuged at 13,000 g for 10 minutes. The supernatant was removed and the cell pellet was stored at -20 °C until further use. The cells in the pellet were re-suspended in 200 μl of digestion solution (Tris 1 mM, 200 μg/ml of proteinase K and 0.5% sodium dodecyl sulfate, SDS). This suspension was shaken and incubated at 55 °C for two hours and at 95 °C for five minutes. Next, 200 μl of solution phenol/chloroform/isoamyl alcohol (25:24:1) was added and shaken vigorously before centrifugation at 5,000 g for 10 minutes. The aqueous phase was removed and transferred to a clean tube, and 1/10 (10%) of NaAc (sodium acetate) 3M pH 5.2 was added and mixed. Next, 2.5 volumes of 70% ice-cold ethanol was added and shaken. The solution was centrifuged at 15,000 g for 15 minutes and the supernatant was removed. After the pellet of deoxyribonucleic acid (DNA) had dried, it was dissolved in 100 μl of Tris-ethylenediaminetetraacetic acid (TE-EDTA) solution (Tris 1 mM, EDTA 100 μM, pH 8.2). The nucleic acids were stored at -20 °C until use.

### Roche Linear Array®

The Roche Linear Array® (LA) HPV genotyping assay (Roche Molecular Systems, Alameda, California, United States) is based on polymerase chain reaction (PCR) amplification of the target DNA using HPV primers (PYGM09/11), hybridization of the amplified product using oligonucleotide probes and then their detection using a colorimetric reaction. The master mix contains primers for amplification of a 450-base pair (bp) fragment of the L1 region of more than 37 HPV genotypes^18^ and a 268-bp fragment of the human b-globin gene, as an internal control.

**Figure 1.**
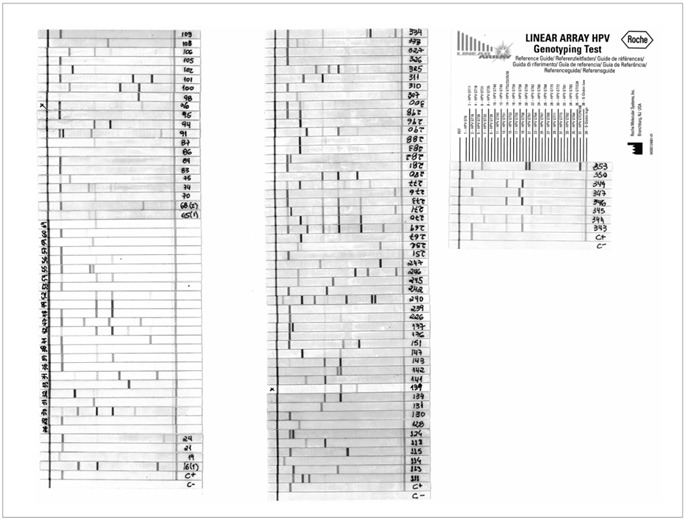
Linear array strips for all cases, aligned with the reference card.

Detection and genotype determination were performed using the denatured amplified DNA and an array of oligonucleotide probes located in the polymorphic region of L1. This enabled independent identification of 37 individual HPV genotypes (Figure 1), as follows: 16 high-risk HPV types (16, 18, 31, 33, 35, 39, 45, 51, 52, 56, 58, 59, 66, 68, 73 and 82), 11 low-risk HPV types (6,11, 40, 42, 54, 61, 70, 72, 81, CP6108 and 67), two intermediate-risk HPV types (26 and 53) and eight HPV types of undetermined risk (55, 62, 64, 69, 71, 83, 84 and IS 39).

### Statistical methods

The data were tabulated in OpenOffice spreadsheets and analyzed in the R environment for statistical analyses. The proportions of CIN 2 and CIN 3 in groups of women infected with various A7 and A9 phylogenetic HPV groups were compared using Fisher’s exact test. Three situations were considered: women with single infections; multiple infections; and the whole sample. A linear regression model was then fitted to assess the proportions of CIN 2 and CIN 3 in groups of women with different single or multiple combinations of infecting HPV types.

## RESULTS

The distribution of HPV types as single and multiple infections is shown in Table 1. Of the 37 HPV types included in LA-HPV, 32 were detected in the samples analyzed. HPV 16 was the most prevalent genotype (present in 57.1% of the samples), either as a single infection or co-infecting the cervix together with other HPV types. It was the only infecting type in 15.2% of the samples. Other frequent genotypes, in either multiple or single infections, were HPV 58 (26/105; 24.7%), HPV 33 (16/105; 15.2%), HPV 52 (14/105; 13.3%), HPV 31 (11/105; 10.4%) and HPV 51 (8/105; 7.6%), which are all high-risk HPV types. HPV 18 appeared in only 6.6% of the lesions, and it was not a single agent in any of them. Only 3/105 (2.87%) of the women had infections composed exclusively of low and intermediate-risk HPV types (as either single or multiple types). The precise composition of the various multiple HPV infections is shown in Figure 1.

**Figure 2.**
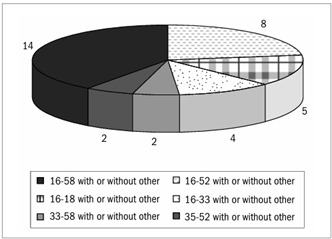
Most prevalent associations of human papillomavirus (HPV) types in multiple infections.

Multiple infections were detected in 68/105 (64.7%) of the women. The majority of these multiple-type infections included two HPV types (39/68; 57.3%). Considerably less frequently, there were three types (17/68; 25%), four types (8/68; 11.7%), five types (2/68; 2.9%) and even six types (2/68; 2.9%) (data not shown). Among the multiple-type infections, 42/68 (61.7%) consisted exclusively of high-risk HPV types. The predominant multiple-type associations (Figure 2) were HPV 16/58 with or without other types (n = 14), followed by HPV 16/52 with or without other types (n = 8), HPV 16/18 with or without other types (n = 5), HPV 16/33 with or without other types (n = 4) and HPV 33/58 and HPV 35/52 with or without other types (n = 2, each).

The distribution of HPV types in accordance with their phylogenetic species (genus Alpha, species 7: HPV 18, 39, 45, 59, 68, 70 and 85; and genus Alpha, species 9: HPV 16, 31, 33, 35, 52, 58, and 67) in CIN 2 and CIN 3 lesions is shown in Table 2. The majority of the women had an HPV type of species Alpha 9 and others, while very few had Alpha 7 and others (5/105). There were 22 women (22/105) with both species (Alpha 9 and Alpha 7). Proportionally, women not infected with HPV species Alpha 9 were less likely to have CIN 3, compared with their Alpha 9 HPV-infected counterparts (P = 0.01). This tendency was also found when analyzing the subset of women with multiple infections only (P = 0.001). In women infected with a single HPV type, the proportion of CIN 3/CIN 2 was the same, regardless of the HPV species present in their cervix (P = 0.20).

The overall detection of HPV by LA was 105/106 women (99%); for CIN 2 it was 100% and for CIN 3 it was 99%. The HPV 16 and 18 single infections, HPV 16/18 co-infections and HPV single or multiple infections without HPV 16 and 18 are summarized in Table 3. Infections including HPV 16 and 18 singly or in association with other HPV genotypes were significantly more common in CIN 3 than in CIN 2 (P = 0.01).

**Table 1.** Distribution of human papillomavirus (HPV) genotypes in single and multiple infections in Brazilian women

HPV genotype	Overall prevalence of HPV genotypes (single and multiple infections) n (%)	Single infections n (%)
High risk		
16	60 (57.1)	16 (15.2)
58	26 (24.7)	6 (5.7)
33	16 (15.2)	4 (3.8)
52	14 (13.3)	1 (0.9)
31	11 (10.4)	2 (1.9)
51	8 (7.6)	1 (0.9)
18	7 (6.6)	0
68	7 (6.6)	0
35	6 (5.7)	1 (0.9)
45	5 (4.7)	0
56	4 (3.8)	1 (0.9)
39	3 (2.8)	0
59	3 (2.8)	0
66	3 (2.8)	1 (0.9)
82	3 (2.8)	0
73	2 (1.9)	0
Intermediate risk		
53	2 (1.9)	0
26	0	0
Low risk		
70	5 (4.7)	1 (0.9)
CP6108	5 (4.7)	0
61	4 (3.8)	0
67	3 (2.8)	0
81	3 (2.8)	0
54	2 (1.9)	0
6	2 (1.9)	1 (0.9)
11	1 (0.9)	1 (0.9)
40	1 (0.9)	0
42	1 (0.9)	0
72	1 (0.9)	0
Not classified		
71	5 (4.7)	0
55	2 (1.9)	0
62	2 (1.9)	0
84	2 (1.9)	0
64	0	0
69	0	0
83	0	0
IS39	0	0

**Table 2.** Distribution of human papillomavirus (HPV) genus A, species 7 and 9 genotypes in cervical intraepithelial neoplasia (CIN 2 and CIN 3) among Brazilian women

Species in sample	Single n (%)		Multiple n (%)		Total n (%)	
	CIN 2	CIN 3	CIN 2	CIN 3	CIN 2	CIN 3
A9 and others (except A7)	1 (20)	29 (88)	8 (61)	34 (62)	9 (50)	63 (72)
A7 and others (except A9)	2 (40)	0	1 (8)	2 (4)	3 (17)	2 (2)
A9 and A7	0	0	3 (23)	19 (34)	3 (17)	19 (22)
Only others	2 (40)	4* (12)	1 (8)	0	3 (17)	4 (4)
Total	5 (100)	33 (100)	13 (100)	55 (100)	18 (100)	88 (100)
	P = 0.20		P = 0.001		P = 0.01	

A9 including 16, 31, 33, 35, 52, 58 and 67; A7 including 18, 39, 45, 59, 68, 70 and 85 *One CIN 3 HPV was HPV negative according to the Linear Array® Comparing CIN 2 and CIN 3 and infection including A7 or A9; P = 0.01

**Table 3.** Occurrence of human papillomavirus (HPV 16 and HPV 18) in cervical intraepithelial neoplasia (CIN 2 and CIN 3) in Brazilian women

HPV Infection	Histological grade	
	CIN 2 n (%)	CIN 3 n (%)
Multiple infections including HPV 16 and/or 18	6 (33)	40 (45)
Single infections including HPV 16 and/or 18	0	16 (19)
Multiple infections including neither HPV 16 nor 18	7 (39)	15 (17)
Single infections including neither HPV 16 nor 18	5 (27)	16 (19)
Total	18 (100)	87 (100)

One CIN 3 HPV was HPV negative according to the Linear Array® (not included in the table)

## DISCUSSION

In this study using the Roche LA-HPV genotyping assay, HPV was detected in 99% of CIN 2 and CIN 3 samples. Similar results were reported by Castle et al.^19^ and Gargiulo et al.^20^ LA-HPV is a commercial HPV test, manufactured following good manufacturing practices with standardized reagents.^19^ In this assay, the same amplicon can be directly used for both detection of b globin and 37 different HPV genotypes in a single reaction. In the present series, this method was easy to use and highly accurate for detecting the majority of clinically significant HPV genotypes.

Unlike DNA sequencing, LA-HPV is capable of identifying multiple infections.^21^^,^^22^ In the present series, 64.7% of the samples had multiple HPV genotypes. This high prevalence of multiple infections is evident in most studies using LA-HPV, ranging from 49.7% to 79.0%.^20^^,^^21^^,^^22^ Detection and genotyping of HPV becomes more complex in samples containing multiple genotypes, because of competition for reagents during amplification and discrimination of the types amplified by PCR. The correlation between the numbers of additional types detected with LA-HPV suggests that less competition during amplification was encountered with LA-HPV.^23^

One clear advantage of full HPV genotyping is the ability to increase the performance of screening programs using HPV, while identifying the HPV-positive women who have persistent oncogenic HPV. There is some recent evidence that the risk of developing the lesion increases with the cumulative number of HPV types, and these associations seemed particularly strong during the first year of follow-up.^16^ Thus, detection of short-term HPV persistence may increase the specificity of the screening based on HPV. If full genotyping is introduced into the screening, the question of how often women who repeatedly test positive for oncogenic HPV (with or without HPV 16 and HPV 18) present persistent oncogenic HPV infection can be resolved.^24^ However, it should be emphasized that current guidelines on cervical screening suggest that HPV testing should be used at intervals no shorter than three years, which offsets the applicability of HPV genotyping, in the form described above.

In this series of Brazilian women, the overall prevalence of HPV 16 was 57.1%, although HPV 16 as a single type was present only in 15.2% of the cases. The associations most frequently found were of HPV 16 together with HPV 58, 52, 18 and 33. In a study very similar to the present one, in terms of design, Prétet et al.^25^ examined a sample of CIN 2 and 3 and found that HPV 16 was the most frequent type. However, in contrast to the present findings, that type was the only agent in 40.4% of the samples. In a recent meta-analysis, Smith et al.^8^ found that the prevalence of HPV 16 (both single and co-infections) in high-grade lesions collected from all around the world was 45.3%.

In our study, the HPV 18 genotype appears in seventh position in terms of prevalence, occurring in 6.6% of the samples. Invariably, HPV 18 was associated with other HPV genotypes. This is very similar to the report by Prétet et al.,^25^ with HPV 18 prevalence of 4.3% (6.6% of 121 CIN 2 lesions and 3.5% of 372 CIN 3 lesions) and only three CIN 3 lesions presented HPV 18 as the single type of infection. In the report by Smith et al.,^8^ HPV 18 appears in fifth position with an overall prevalence of 6.9%. There are some minor differences between this meta-analysis^8^ and our results, regarding the order of prevalence of the most common HPV genotypes: 16, 31, 33, 58, 18, 52, 35 and 51 in the meta-analysis; and 16, 58, 33, 52, 31, 51, 18 and 68 in the present series. However, these authors stated that there was an important lack of South American data in their meta-analysis, and our present study adds new information concerning the HPV type distribution in high-grade lesions among Brazilian women.

Comparing CIN 2 and CIN 3, HPV 16 and 18 were significantly more prevalent in CIN 3 lesions. This is consistent with the data of Prétet et al.,^25^ who analyzed 121 CIN 2 and 372 CIN 3 lesions in France, and also found higher prevalence of HPV 16 in CIN 3 (64.2%) than in CIN 2 lesions (56.2%). This difference was even more marked in a study by Zuna et al.,^9^ who reported HPV 16 infection in 65.6% of CIN 3 and only 19.0% in CIN 2 lesions. Despite the fact that in all reported series, CIN 2 lesions represented a minority of cases, these data suggest that CIN 2 and 3 might differ in their biological potential, and that HPV genotypes might interfere with the risk of progression in these two categories of CIN lesions.

According to the results from the present study, women infected with Alpha 9 species were more likely to have CIN 3 than were women infected with Alpha 7 species and others. These data are compatible with the information on the carcinogenic potential of specific HPV types. In fact, studies have demonstrated that HPV 16, 18 and 45 are more prevalent in squamous cell carcinoma than in HSIL (CIN 2 and CIN 3), whereas the reverse is true for other oncogenic types, including HPV 31, 33, 52 and 58. Infection with HPV 16 imposes the highest risk of both CIN 3 and cervical carcinoma, although HPV 33, which also belongs to the Alpha 9 species, possibly has a greater oncogenic potential than HPV 18 and 45.^7^ In another epidemiological study, Wheeler et al.^26^ observed a higher risk of CIN 3 among women with HPV 31 (Alpha 9) and relatively low oncogenic potential for HPV 56 and 59 (Alpha 7).

The present study provides new information on the distribution of individual HPV genotypes and multiple infections, obtained using the Roche LA-HPV assay. These data suggest that a prophylactic vaccine against HPV 16/18 has the potential to prevent approximately half of the high-grade lesions. For one of the two candidate vaccines, Harper et al.^27^ stated that this proportion might even be increased, if cross-protection were achieved against some other high-risk HPV types, e.g. total protection against HPV 45, partial protection against HPV 31 and no protection against HPV 33, 52 and 58. However, the full implications of this observation are difficult to define, because HPV 45 was under-represented in our cohort, in the same way as in the recent meta-analysis.^8^ These data on HPV genotype distribution among different populations are of crucial importance for designing second-generation prophylactic HPV vaccines in the near future. Also of relevance, future therapeutic HPV vaccines would have greater potential benefit if their design for treating HPV infections were not restricted to HPV 16 and 18. The best hypothetical scenario is one in which vaccine constituents are chosen taking into consideration the epidemiological distribution of HPV types in the specific geographical regions where they are to be used.

## CONCLUSIONS

The present series firmly corroborates the assumption that, in most CIN 2 and CIN 3 cases, multiple high-oncogenic HPV types may be found. In our series, the prevalence of HPV 58 and HPV 33 was unexpectedly high. CIN 3 was typically associated with HPV genus Alpha, species 9, most often consisting of types 16 and 58, alone or in combination with other HPV types.
